# The miRNA-Mediated Post-Transcriptional Regulation of Maize in Response to High Temperature

**DOI:** 10.3390/ijms20071754

**Published:** 2019-04-09

**Authors:** Moubiao Zhang, Panpan An, Hongping Li, Xiuling Wang, Jinlong Zhou, Pengfei Dong, Yali Zhao, Qun Wang, Chaohai Li

**Affiliations:** College of Agronomy, Henan Agricultural University, Zhengzhou 450000, China; zhangmoubiao@126.com (M.Z.); anpanpan2018@163.com (P.A.); lmhlhp021506@163.com (H.L.); xiuling845697624@163.com (X.W.); 18537700566@163.com (J.Z.); 13783638412@163.com (P.D.); zhaoyali2006@126.com (Y.Z.); wangqun177@163.com (Q.W.)

**Keywords:** maize, high temperature, miRNA, transcriptome, degradome

## Abstract

High temperature (HT) has recently become one of the most important abiotic stresses restricting crop production worldwide. MicroRNAs (miRNAs) are important regulators in plant development and stress responses. However, knowledge of miRNAs of maize in response to HT is limited. In this study, we simultaneously adopted miRNA sequencing and transcriptome profiling to analyze the differential expression of miRNAs and mRNAs in maize during exposure to HT stress. Our analysis revealed 61 known miRNAs belonging to 26 miRNA families and 42 novel miRNAs showing significant differential expression, with the majority being downregulated. Meanwhile, the expression of 5450 mRNAs was significantly altered in the same stressed tissues. Differentially expressed transcripts were most significantly associated with response to stress, photosynthesis, biosynthesis of secondary metabolites, and signal transduction pathways. In addition, we discovered 129 miRNA–mRNA pairs that were regulated antagonistically, and further depiction of the targeted mRNAs indicated that several transcription factors, protein kinases, and receptor-like-protein-related transmembrane transport and signaling transduction were profoundly affected. This study has identified potential key regulators of HT-stress response in maize and the subset of genes that are likely to be post-transcriptionally regulated by miRNAs under HT stress.

## 1. Introduction

Maize (*Zea mays* L.) is one of the world’s three most important cereal crops, along with rice and wheat, and holds a prominent position in the world’s agriculture. High temperature (HT) has recently become one of the most critical abiotic stresses restricting maize production worldwide [1], including in the United States [2], France [3], and Africa [4]. The Yellow and Huai River valleys, which constitute one of the main summer maize production belts in China, also frequently encounter heat stress at almost all growth stages, which has led to severe yield loss [5]. In addition, a 1 °C increase in the growing-season average temperature has been estimated to result in a loss of more than 10% of the maize yield due to frequent exposure to temperatures above 30 °C [4]. Moreover, the daily mean temperature was predicted to increase by approximately 2.0–3.7 °C by the end of the 21st century, and this increase will likely be accompanied by an increased frequency of heat waves [6]. Therefore, how to cope with HT stress is an urgent issue that needs to be solved.

To alleviate the adverse effects of HT stress on crop growth and development, it is necessary to first address the underlying mechanism used by crops to cope with HT stress. HT stress can induce physiological, molecular, and biochemical changes that disturb various cellular and whole-plant processes, which in turn negatively influence the development and yield of crops. Some examples include the following: cell membranes might become disorganized, osmotic homeostasis could be altered, proteins might lose activity or be denatured, and the levels of reactive oxygen species (ROS) could increase and result in oxidative damage [7]. As sessile organisms, plants need an efficient strategy, such as the modulation of gene expression, to adapt and survive under abiotic stress conditions.

MicroRNAs (miRNAs) are a recently discovered class of endogenous noncoding small RNAs that serve as ubiquitous critical regulator molecules by negatively modulating gene expression at the posttranscriptional level by either targeting mRNAs for cleavage or inhibiting their translation based on the extent of the complementarity between the miRNA and its target [8]. High-throughput sequencing is a powerful tool for discovering differentially expressed genes (DEGs) in the whole genome and is especially useful for studying complex gene regulatory networks [9]. Some HT-responsive miRNAs in several plants have been detected through miRNA sequencing [10], and the HT-responsive mRNAs of rice [11,12], barley [13], chili pepper [14], and maize [15,16] have also been evaluated by mRNA sequencing. It is inspiring that a few transgenic studies have further proved the vital role of miRNAs in plant abiotic stress tolerance. For example, overexpressing miR156 enhanced *Arabidopsis* tolerance to HT stress [17], overexpressing miR169 significantly enhanced tomato tolerance to drought stress [18], and overexpressing miR157 and miR160 increased cotton sensitivity to HT stress [19]. Thus, miRNA is promising for use in the abiotic stress tolerance improvement of crops. Maize is one of the most important crops suffering from HT stress worldwide and thus an important model organism for studies in plant genetics, physiology, and development, distinguished from other plants by its large and complex genome (about 2.3 G) and the C4 pathway. However, the miRNAs of maize in response to HT stress have not yet been clarified. Moreover, the previous transcriptome profiling-based studies of miRNAs or mRNAs in response to HT stress are independent, i.e., the extracted miRNA or mRNA used for sequencing in the different studies are not from the same tissue or collected at the same sampling time, even if the study was focused on the same species. Because miRNAs and mRNAs show spatiotemporal expression, an integrated analysis of miRNA and mRNA sequencing that was simultaneously performed is needed to obtain a proper understanding of the regulatory action of miRNAs under HT stress. However, no such combined analysis has been performed.

In this study, we simultaneously performed miRNA sequencing, degradome sequencing, and transcriptome profiling with two aims: (1) to investigate the HT-responsive miRNAs of maize, (2) to analyze the changes in mRNA profiles associated with miRNA regulation in maize exposed to HT stress. Integrated analysis of mRNA and miRNA datasets uncovered the miRNA-mRNA interaction pairs involved in specific biological processes of maize during exposure to HT stress. Our results provided valuable information with regard to revealing the miRNA-mediated post-transcriptional regulation of maize in response to HT stress.

## 2. Results

### 2.1. Phenotypic and Physiological Responses of Maize Leaves to HT Stress

**Figure 1:**
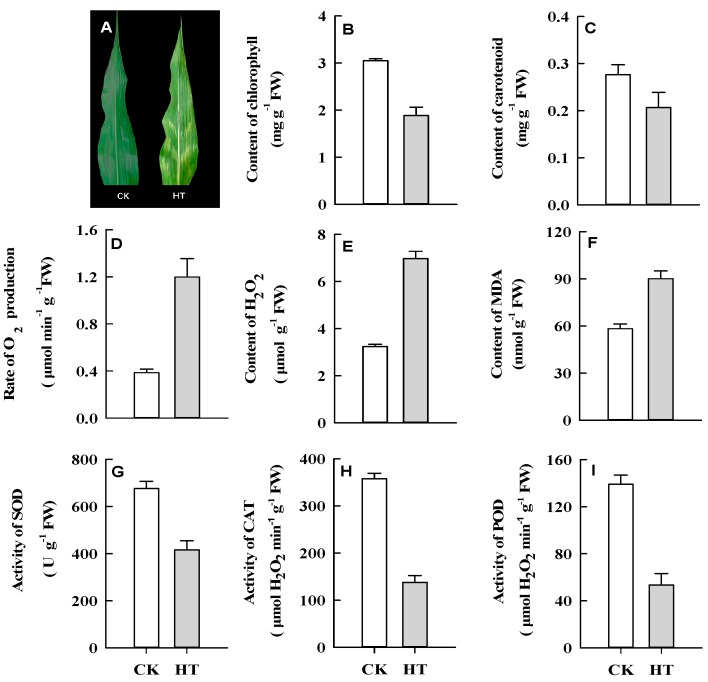
Effects of high temperature on phenotype (**A**), photosynthetic pigments (**B**,**C**), reactive oxidative species (**D**,**E**), lipid peroxidation (**F**) and antioxidase (**G**–**I**) of maize leaves.

HT stress had a marked impact on maize leaf growth. In our study, obvious heat injury was observed in maize leaves after five days of exposure to HT, and this symptom was very prominent in the 10th leaf of stressed plants (Figure 1A). For this reason, we harvested the 10th leaves of planted maize after five days of exposure to the control (CK) and HT conditions for physiological parameter determination and further RNA-seq profiling. As expected, the content of photosynthetic pigments in leaves showed an obvious decline under HT (Figure 1B,C). Moreover, ROS production and accumulation and membrane lipid peroxidation showed significant increases, as indicated by the production rate of superoxide anion, the H_2_O_2_ level, and the malonaldehyde (MDA) content, respectively (Figure 1D,E), and the antioxidase activity (Figure 1G–I) of maize leaves was significantly reduced under HT stress. All these results indicated that HT stress exerted negative effects on the growth and development of maize leaves.

### 2.2. Analysis of Small RNA-seq Data

**Figure 2:**
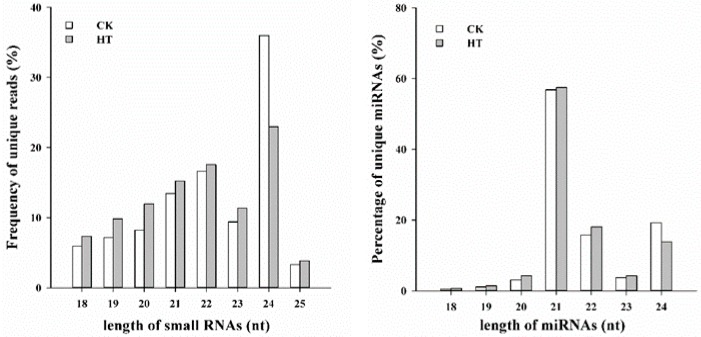
Size distribution of unique small RNAs and miRNAs detected by deep sequencing.

To determine the involvement of regulatory miRNAs in adaptation to high temperature, six small RNA libraries were constructed from maize leaves exposed to CK and HT conditions and sequenced by Illumina NGS technology. This analysis yielded 9.9, 10.8 and10.3 M raw reads from the CK samples and 10.9, 12.8 and 12.6 M from the HT samples, respectively. After removing low-quality reads and adaptors, an average of 567,792 and 4,990,915 valid reads ranging from 18 to 25 nt in size were retained for the construction of CK and HT small RNA libraries, respectively (Table S1). In these libraries, unique small RNAs of 24 nt were the most abundant, followed by RNAs of 22 and 21 nt, respectively (Figure 2).

### 2.3. Identification of Known and Novel Differentially Expressed miRNAs (DEMs)

To identify known and novel miRNAs in CK and HT libraries, the unique clean reads were aligned to pre-miRNAs of selected species in the miRbase. A total of 223 known miRNAs were identified in small RNA libraries from the CK and HT samples (Table S2). The remaining sequences that did not match any of the known miRNAs or pre-miRNAs were then further analyzed based on the criteria for annotation of plant miRNAs [20], and 261 novel miRNAs were identified (Table S3).

**Figure 3:**
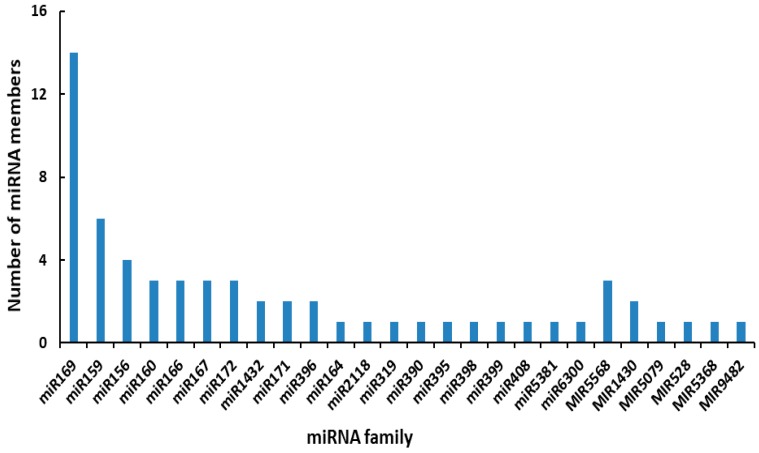
Number of DEMs member in each miRNA family.

To identify DEMs responsive to HT stress, we compared the expression values of miRNAs in CK and HT libraries using Student’s *t*-test. Of the 223 identified known miRNAs, 61 miRNAs were differentially expressed (*p* < 0.05) (Table S4). Among the known DEMs, the miR169 family had the highest number of members, followed by the miR159, miR156, and miR160 families; the majority of these families had fewer than 10 members (Figure 3), suggesting that differential expression of different miRNA genes belonging to the same family contributed to the final number of miRNAs produced in the maize leaves. The results indicated that the majority of the DEMs (52 of 61) were downregulated. Among the 261 identified novel miRNAs, 42 were differentially expressed (*p* < 0.05). Similarly, all novel DEMs, with the exception of miRn218, which had log_2_FC values of 3.4 (*p* = 0.01), were downregulated (Table S5).

### 2.4. Target Prediction and Functional Annotation of the Known and Novel DEMs

To identify the target genes of the known and novel DEMs identified in this study, miRNA target prediction and degradome sequencing were performed. A total of 1073 transcripts (predicted and sequenced) from 368 genes were predicted to be targets of 52 known DEMs (Table S4). A total of 273 transcripts from 80 genes that are targets of 31 known DEMs were then further confirmed by degradome sequencing using CleaveLand3.0 pipeline. For novel DEMs, a total of 1176 transcripts from 422 target genes were predicted to be targets of 40 novel DEMs. However, only 37 transcripts from 13 target genes for 10 novel DEMs were identified in the degradome library (Table S5). The results showed that many target genes regulated by known miRNAs were members of different families of transcription factors, including the AP2, ARF, GRF, HD-ZIP, NAC, NF-YA, and SBP families (Table 1). A gene ontology (GO) analysis of the target genes of known and novel DEMs was performed to shed light on their biological functions. This analysis showed that these target genes were related to transcription, developmental process, proteolysis, lipid binding and metabolism, signaling transduction, and oxidation–reduction functions, among others (Table 2 and Table 3).

### 2.5. HT-Responsive Transcriptome in Maize Leaves

**Figure 4:**
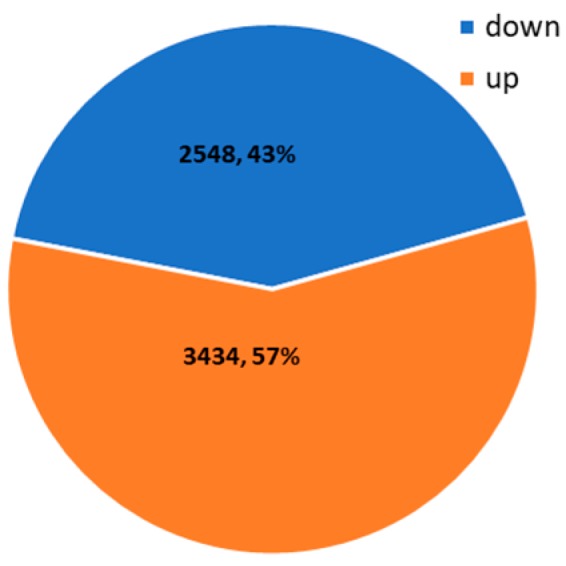
Number of differentially expressed genes (DEGs) under control and HT conditions.

To detect the mRNA expression profiles of maize in response to HT stress, RNA-seq was performed using the same RNA samples that were previously used for small RNA-seq of maize under CK and HT conditions. After removing the low-quality reads and trimming off the adapter sequences, approximately 42,693,136~50,558,368 and 46,887,354~52,075,584 reads were obtained for CK and HT samples, respectively. Approximately 49.05%~61.49% and 55.20%~58.53% of the reads obtained from the CK and HT samples, respectively, were uniquely mapped to the maize reference genome. An overview of the read information is shown in Table S6. Finally, 5982 genes with an absolute value of log_2_FC ≥ 1 and *p*-value < 0.05 were identified as DEGs, and these included 3434 upregulated and 2548 downregulated genes (Table S7 and Figure 4). To dissect their potential functions, these DEGs were subjected to further GO and KEGG enrichment analyses.

**Figure 5:**
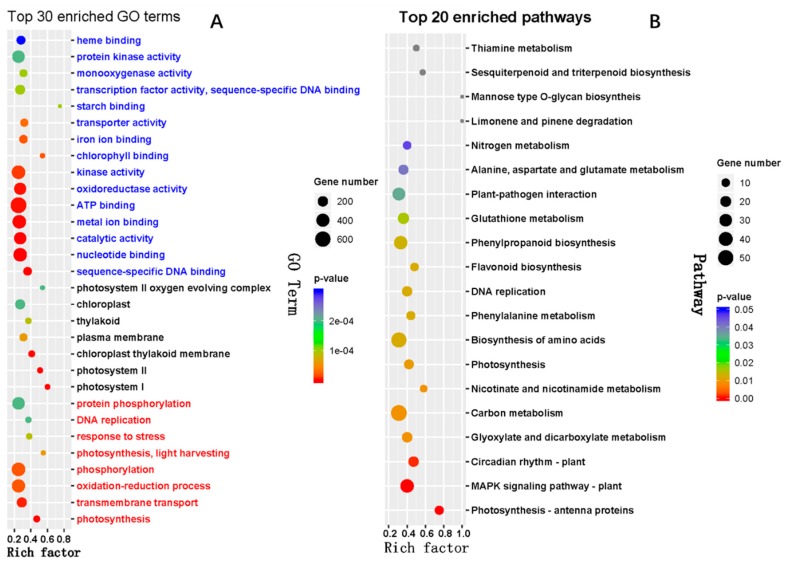
GO (**A**) and KEGG (**B**) functional classifications of differentially expressed genes (DEGs). Rich factor represents the ratio of the number of DEGs over the total number of genes in the specific GO term or KEGG pathway. The GO terms in red, black, and blue belong to biological processes, cellular components, and molecular functions, respectively.

The GO enrichment analysis revealed that the 30 most significantly enriched GO terms were assigned to eight, seven, and 15 GO terms in the biological process, cellular component, and molecular function ontology categories, respectively (Figure 5A). The GO terms belonging to biological process included photosynthesis/GO:0015979, transmembrane transport/GO:0055085, oxidation-reduction process/GO:0055114, phosphorylation/GO:0016310, photosynthesis, light harvesting/GO:0009765, response to stress/GO:0006950, DNA replication/GO:0006260 and protein phosphorylation/GO:0006468. The most enriched GO terms related to cellular component included photosystem I/GO:0009522, photosystem II/GO:0009523, chloroplast thylakoid membrane/GO:0009535, plasma membrane/GO:0005886, thylakoid/GO:0009579, chloroplast/GO:0009507, and photosystem II oxygen evolving complex/GO:0009654, which indicated that the structure of the chloroplast was severely affected during exposure to HT stress. Among the three abovementioned ontologies, the highest number of DEGs and GO terms were associated with molecular function, particularly ATP binding/GO:0005524, metal ion binding/GO:0046872, kinase activity/GO:0016301, nucleotide binding/GO:0000166, protein kinase activity/GO:0004672, catalytic activity/GO:0003824, oxidoreductase activity/GO:0016491 and transcription factor activity (sequence-specific DNA binding)/GO:0003700.

To elucidate the exact biological process that the DEGs might participate in, we performed a KEGG enrichment analysis, which revealed that 16 vital pathways were significantly enriched (Figure 5B). The results clearly showed that plant energy metabolism was affected due to heat-induced leaf injury, as revealed by the finding that the most significantly enriched pathways were photosynthesis pathways (zma00195) and nitrogen metabolism (zma00910), particularly photosynthesis-antenna proteins (zma00196). In addition, the genes involved in carbohydrate metabolism were enriched in carbon metabolism (zma01200) and glyoxylate and dicarboxylate metabolism (zma00630), the genes participating in environment adaptation were enriched in circadian rhythm-plant (zma04712) and plant-pathogen interaction (zma04626), the genes related to amino metabolism were enriched in amino acid biosynthesis (zma01230), phenylalanine metabolism (zma00360), alanine, aspartate and glutamate metabolism (zma00250) and glutathione metabolism (zma00480), and the genes taking part in secondary metabolite biosynthesis were enriched in flavonoid biosynthesis (zma00941), phenylpropanoid biosynthesis (zma00940) and nicotinate and nicotinamide metabolism (zma00760). Moreover, other significantly enriched pathways, including DNA replication (zma03030) and MAPK signaling pathway in plants (zma04016), contribute in the regulation of plant hormone signaling and plant defense during exposure to HT stress.

### 2.6. Target mRNAs Negatively Regulated by DEMs

Based on sequence complementarity, 4515 miRNA-mRNA pairs were predicted by TargetFinder, and 3059 of these pairs could be detected in our transcriptome analysis. we subsequently filtered out 280 miRNA-mRNA pairs in which mRNAs showed significant differential expression according to our mRNA transcriptome data. However, it should be noted that we only considered those genes that are likely to be cleaved by miRNAs; the translational inhibition of gene expression by miRNAs is also important but could not be considered in the present analysis. The study revealed that 129 of 280 miRNA-mRNA pairs showed the opposing expression patterns under HT conditions (Table S8).

**Figure 6:**
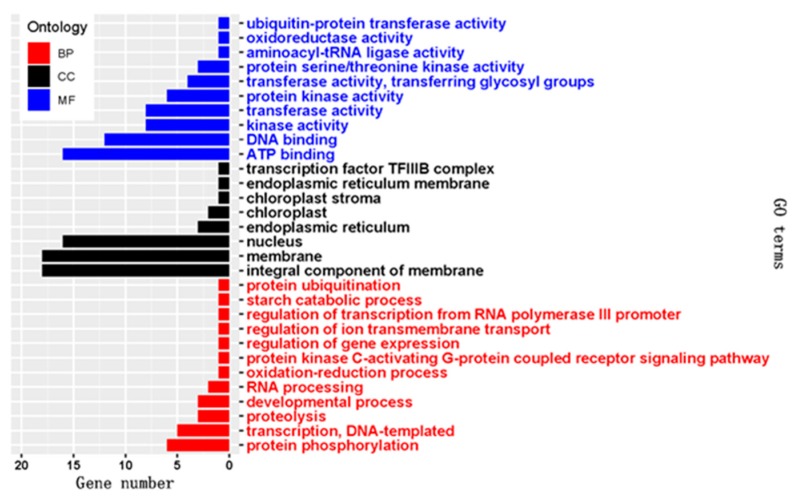
The GO terms in red, black, and blue belong to biological processes, cellular components, and molecular functions, respectively, enriched by target mRNAs negatively regulated by DEMs.

There were 13 miRNA-mRNA pairs consisting of eight DEMs and 12 oppositely regulated mRNAs from genes that participate in important pathways, including zma00561 (glycerolipid metabolism), zma04070 (phosphatidylinositol signaling system), zma00240 (pyrimidine metabolism), zma00410 (beta-alanine metabolism), zma00770 (pantothenate and CoA biosynthesis), zma04141 (protein processing in ER), zma04120 (ubiquitin-mediated proteolysis), zma00230 (purine metabolism), zma00970 (aminoacyl-tRNA biosynthesis), zma00860 (porphyrin and chlorophyll metabolism), zma04144 (endocytosis), and zma03040 (spliceosome) (Table 4). Most negatively regulated targets were enriched in GO terms related to integral components of the membrane, nucleus, ATP binding, and DNA binding. Genes related to the oxidation-reduction process, chloroplast, regulation of transcription, and protein phosphorylation and proteolysis were also enriched (Figure 6).

Furthermore, a degradome analysis of maize confirmed 30 of these interactions, which comprised 12 miRNAs mainly belonging to the miR156, miR166, miR169, miR172, miR396, and miR5381 families, and 17 negatively regulated mRNAs from 12 genes. Most of these targets (18/30) are transcription factors with roles in stress-related genes or pathway regulation (Table S9), which suggests that the miRNA-mediated regulation of the transcriptome is an essential process in the response of maize to HT.

### 2.7. Quantitative Real-Time PCR Validation

**Figure 7:**
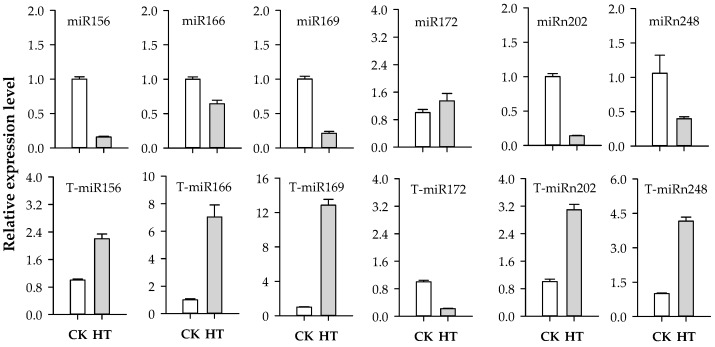
Confirmation by qRT-PCR of miRNAs and target genes identified in sequence data. Bars indicate the standard error of three replications.

To validate the deep-sequencing data, the expression of miRNAs and mRNAs was analyzed by quantitative real-time PCR (qRT-PCR). Six miRNAs, including four known miRNAs and two novel miRNAs, and their corresponding targets were selected for qRT-PCR analysis (Figure 7 and Table S10). These results were consistent with that of analysis of sequencing data for miRNAs and the transcriptome.

## 3. Discussion

HT is a detrimental abiotic stress that affects the growth duration, growth pattern and productivity of crops, and many studies have attempted to elucidate the strategy used by crops to adapt to a high-temperature environment [12,15,16,21,22]. The identification of miRNAs and their targets will lay the foundation for unraveling the complex miRNA-mediated regulatory networks controlling the response to HT stress. However, the miRNAs of maize in response to HT stress and the mRNAs that are precisely regulated by miRNAs during HT at the whole genome level have not been identified. In this study, to determine why heat-responsive genes were differentially expressed in maize under HT stress and thus to provide an enhanced understanding of the potential miRNA regulatory network under HT, we performed a multi-omics high-throughput sequencing analysis of the expression of miRNAs and mRNAs in the same sample.

### 3.1. miRNAs of Maize in Response to High Temperature (HT)

The overall analysis of small RNA data revealed that the sRNA length distribution patterns showed a peak at 24 nt (Figure 2), which is consistent with previous results for most plants, such as radishes [23], tomatoes [24], wheat [25], rice [26], and cotton [19]. Therefore, the sRNA length distribution pattern in maize is similar to that in other crops. Interestingly, the total read number of 21-nt sRNAs increased, while 24-nt population decreased markedly under HT stress (Figure 2). miRNAs or secondary siRNAs are 21 nt in length, whereas the heterochromatic siRNAs are 24 nt in length and play a possible role in DNA methylation [27]. Thus, it will be interesting to further investigate whether HT stress has led to a changed methylation level at some specific loci, which in turn resulted in the expression of HT responsive genes.

In this study, 61 known and 41 novel miRNAs were found be differentially expressed (Tables S4 and S5). These were assigned to their family based on the sequence similarity among miRNAs, and most miRNA members within the same family showed a similar expression pattern, which is consistent with previous observations [23,24,28]. For instance, four miR156 and 14 miR169 family members were significantly downregulated by HT stress (Table S4). Our results indicated that the majority of these differentially regulated miRNAs were downregulated in maize under HT, and these findings were in accordance with previous studies in other plant species, such as *Populus tomentosa* [18], radishes [23], tomatoes [24], and rice [26], under HT stress, indicating that the downregulation of miRNAs might play a more important role in the regulation of HT-responsive networks. However, in our study, the number of downregulated miRNAs (92) is substantially higher than that of upregulated miRNAs (10), whereas the number of downregulated miRNAs is just a slightly more than that of upregulated in previous studies. Moreover, different miRNA members within the same family presented different expression patterns under HT stress. For instance, zma-miR166c-5p_L-2R+2 and zma-miR166a-3p are significantly downregulated whereas zma-miR166h-5p_L-1R+1 is significantly upregulated. Similar situation was also observed in other plants. In the shoots of a heat-sensitive rice variety, osa-miR169f.2 was downregulated, while osa-miR169e was upregulated under long-term HT stress [26]. Hence, it appears that the regulatory pattern underlying the expression of miRNAs under HT stress is plant-specific, and it is difficult to generalize a rule of regulation of miRNAs during stress due to the different treatment conditions used for different plant species.

### 3.2. Transcriptomics of HT-Stressed Leaves of Maize

Profiling of differentially expressed transcripts under HT stress, and subsequent functional and pathway enrichment analyses provided a comprehensive view of the response regulators during HT stress. Photosynthesis is one of the most heat-sensitive processes [29], and in this study, we found that most of proteins encoded by differentially expressed genes were localized in multiple cellular components, including chloroplast and its structural components and plasma membrane, were enriched in chlorophyll activity, and were involved in multiple biological processes associated with photosynthesis (Figure 5A). Obviously, these genes are closely related to the function of chloroplast, particularly photosynthesis, as was also revealed by KEGG analysis (Figure 5B). Similar finding was also observed in previous studies concerning the transcriptional response of other plants to HT [14,15,30,31], indicating that these altered genes constitute one of major responses of plants to HT stress.

HT stress generally induced oxidative stress, which would lead to peroxidation of membrane lipids and pigments [32], as revealed by the production rate of superoxide anion and the contents of H_2_O_2_ and malonaldehyde (MDA) in our study (Figure 1D,E), which was consistent with a previous study [33]. Moreover, the genes related to oxidoreductase activity and the oxidation-reduction process were also found to be enriched in our analysis (Figure 5A). Some of the other most enriched pathways included secondary metabolism, transcription regulation, circadian rhythm, signaling transduction, and amino metabolism including glutathione, which were also observed in other reports [15,34,35], indicating that these pathways might be the most fundamental pathways involved in heat tolerance in crops.

Our profiling analyses also revealed (i) which KEGG pathways are represented by up- and downregulated genes (Tables S11 and S12), and (ii) the expression status of genes involved in an enriched pathway. These analyses provided an enhanced perspective to the overall expression data. In fact, our results revealed that most enriched biological processes involving only upregulated genes were associated with photosynthesis including photosynthesis-antenna proteins, carbon fixation in photosynthetic organisms and starch and sucrose metabolism, signal transduction including plant hormone signal transduction and the MAPK signaling pathway in plant, lipid metabolism including glycerophospholipid and linoleic acid metabolism and glycosphingolipid biosynthesis–ganglion series, riboflavin metabolism and endocytosis (Table S11). In contrast, most enriched biological processes involving only downregulated genes included protein processing in ER, ribosomal proteins and ribosome biogenesis in eukaryotes, fatty acid metabolism and degradation, biosynthesis of secondary metabolites such as phenylpropanoid, flavonoid and terpenoid, ubiquitin mediated proteolysis and carbon metabolism (Table S12). Moreover, in line with the decreased content of photosynthetic pigments (Figure 1B,C), the genes involved in porphyrin and chlorophyll metabolism and carotenoid biosynthesis were also found to show downregulated expression patterns (Table S12).

The heat signal perception and transduction are essential for plant stress tolerance [36]. Glutathione are known for their ability to protect a plant from oxidative stress and regulate cell proliferation and death by interfering with the MAPK pathway [37]. In our study, the genes related to plant hormone signal transduction and MAPK signaling pathway were upregulated, which might help the activation of stress defense genes and contribute to maize tolerance to HT stress. The phenylpropanoid metabolism pathway generates diverse types of secondary metabolites that can protect plants from abiotic stress. However, genes involved in secondary metabolite biosynthesis pathways including phenylalanine and phenylpropanoid biosynthesis were downregulated, indicating that a decrease in the expression of genes responsible for phenylalanine metabolism might not provide sufficient precursor (phenylalanine) molecules for phenylpropanoid metabolism, which in turn might result in decreased maize tolerance to HT stress in this study.

Interestingly, both upregulated and downregulated genes have been identified to be involved in circadian rhythm in plant and phosphatidylinositol signaling system, indicating that genes involved in these pathways showed either upregulating or downregulating expression patterns, which might help maintain the balance of these pathways under HT stress. Furthermore, although photosynthesis was severely affected under HT stress, which could be inferred from the HT-induced injury in leaves, most of genes related to photosynthesis were upregulated, which appears to be explained by the loss of functional proteins due to the decreased ribosome biogenesis and protein processing in ER. A similar situation was also observed in a recent study [15]. Generally, global inhibition of protein synthesis occurs in response to a number of stress conditions, and protein synthesis is blocked at the level of translation under most of these conditions [38]. To maintain protein homeostasis, cells might increase the expression of many functional genes at transcriptional level; however, this would require the consumption of more energy and resources under long-term stress and might even exacerbate the disturbed homeostasis. In summary, genes involved in several crucial pathways such as activation of signal transduction and metabolic pathways, including the biosynthesis of secondary metabolites, were altered in maize during exposure to HT stress.

### 3.3. Integrated Analyses of miRNA–mRNA Transcriptomics

In general, because miRNAs regulate target gene expression by repressing their targets through transcript cleavage or translation repression, the increased miRNA activity leads to the downregulation of a mRNA target, whereas the decreased miRNA activity leads to upregulation of the target [39,40]. Hence, a transcriptome-wide combined analysis of miRNA and mRNA expression levels was performed to identify the miRNA–mRNA pairs, in which mRNAs were negatively regulated by miRNAs in maize during exposure to HT stress. This analysis enabled us to understand which pathways and biological processes of the cell were most likely regulated by miRNAs under HT stress.

In this study, 4515 miRNA-mRNA pairs were predicted by TargetFinder, and 3059 of these pairs could be detected in our transcriptome analysis. A closer inspection of transcriptomics data showed that 45 miRNAs formed 129 miRNA–mRNA interaction pairs with opposing regulation patterns (Table S8). However, this number is far below that (3059) of the predicted miRNA–mRNA interaction pairs, and possible reasons for this phenomenon include the following: (1) plants can regulate the expression of specific genes at the temporal and spatial levels, and most targets might not be expressed at this point, and (2) the accepted standard used for the definition of DEGs may miss some interactions, and more interaction pairs could potentially be identified by lowering the threshold.

**Figure 8:**
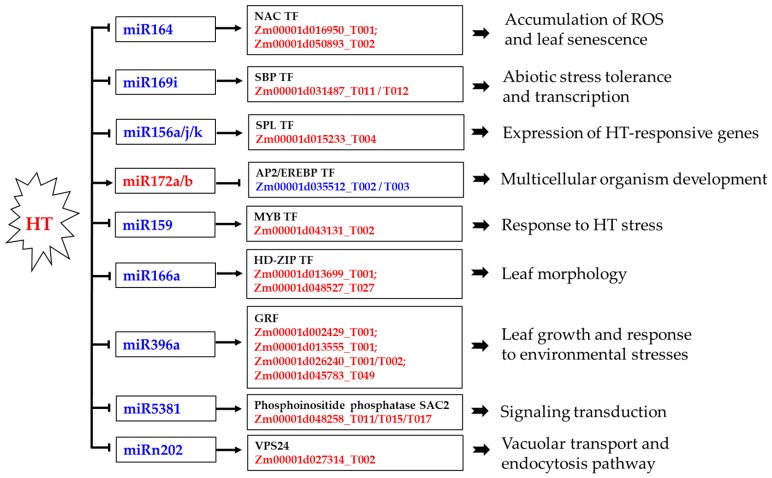
Scheme showing some of the key heat-responsive miRNAs and their targets in maize. The arrows and hammers indicate positive and negative regulation, respectively. The miRNAs/mRNAs in red and blue font were upregulated and downregulated under HT, respectively.

A comparison between the GO functional enrichment of these predicted targets (Table 2 and Table 3) with that of the differentially expressed transcripts (Figure 5A) revealed the biological processes that were likely regulated by miRNAs under HT stress. For instance, several genes related to the term kinase activity and membrane and transmembrane transport were enriched in both analyses. However, no genes related to photosynthesis was enriched among the predicted targets, although photosynthetic pathway was found to be affected as a whole under HT stress, implying that photosynthetic genes are mainly regulated transcriptionally and might not be significantly affected by miRNA-mediated cleavage pathway. These observations suggest that only a few specific biological processes are regulated by miRNAs under HT stress (Figure 8). Even so, we found several important regulatory miRNA-mRNAs involved in HT stress, such as miR156-SBP/SPL, miR169-SBP, miR172-AP2, miR159-MYB, miR164-NAC, miR166-HD zip, miR396-GRF, miR5381-SAC2, and miRn202-VPS24 homolog 1, which can be further confirmed by an analysis of the degradome.

The NAC superfamily is one of the largest families of plant transcription factors (TFs) and is widely distributed in plants [41]. NAC TFs regulate several processes in plants, such as leaf senescence [42], the formation of secondary walls [43], and hormone signaling [44]. Moreover, many members of the NAC TF family coordinate the responses to abiotic stress, including salt [45], drought, and HT [46]. Overexpression of one NAC TF in rice results in an enhanced tolerance to drought and HT stress through the modulation of ROS [46]. In our study, the targets Zm00001d016950_T001 and Zm00001d050893_T002, two NAC TFs, are upregulated by the downregulated zma-miR164f-5p, and the ROSs accumulated in leaves and eventually led to heat injury in maize leaves (Figure 1), indicating that miR164 is also one main regulators of response of maize to HT stress at post-transcriptional level through the modulation of ROS and leaf senescence.

In *Arabidopsis*, squamosa promoter binding (SBP)-box TF genes have been shown to play a role in regulating the rate of leaf initiation [47], flowering time and developmental timing [48]. Moreover, overexpression of one SBP-Box gene in *Arabidopsis* improved salinity and drought stress tolerance [49]. In our study miR169 was downregulated, and two of its targets encoding SBP-transcription factor 16 showed an increased expression level in maize under HT stress, which indicates that a reasonable enhanced defense response of maize to HT stress although the constitutive overexpression of miR169 in transgenic tomato significantly enhanced plant tolerance to drought stress [18].

Plant squamosa promoter binding protein-like (SPL) genes are involved in leaf development, response to stresses and positive regulation of the inflorescence meristem [50]. The miR156 targets SPL to coordinate the relationship between development and abiotic stress tolerance in plants [51]. A recent analysis of miR156-overexpressing *Arabidopsis* revealed that miR156 is required for heat stress memory, and these plants exhibit enhanced HT tolerance [17]. In our study, several upregulated genes encoding SPL are targeted by the downregulated miR156, indicating that the downregulation of miR156 might contribute to the susceptibility of maize to HT, revealed by the heat injury in maize leaves.

The APETALA2/ethylene-responsive element binding protein (AP2/EREBP) superfamily is one of the largest groups of plant-specific transcription factors, which play vital roles in plant growth, development, response to abiotic stress and hormones-related signal transduction pathway [52]. The miR172 functions in the biotic stress response and root development by negatively regulating its target AP2 TF [53]. In our study, the expression of miR172 was upregulated, whereas its target encoding AP2 TF showed the opposite expression pattern, indicating that miR172 might also participate in leaf development under HT stress. Moreover, miR156 acts by repressing the expression of SPL TFs that directly bind to the MIR172 promoter and positively regulate the expression of miR172 in *Arabidopsis* [54]. Transgenic soybean roots overexpressing miR156 showed the decreased expression of two SPL genes and a decreased miR172 level [55], although evidence for the binding of SPL to MIR172 promoters for transcription activation was not provided. In this study, the expression of miR156 is downregulated, whereas that of its target gene encoding SPL and miR172 is upregulated, which suggested that one negative feedback loop, miR156-SPL-miR172, might contribute to maize leaf development under HT stress.

Several recent studies have indicated that the miR159-MYB pathway might also be involved in the abiotic stress response. For instance, the ABA-induced accumulation of miR159 is a homeostatic mechanism to desensitize hormone signaling during seedling drought response and direct AtMYB33 and AtMYB101 transcript degradation [56], and TamiR159-overexpressing rice lines and the *Arabidopsis* myb33-myb65 double mutant are both more sensitive to HT stress than the wild type [57]. In our study, MYB domain protein 81, a MYB transcription factor, is predicted to be one target of zma-miR159, which is also confirmed by our degradome analysis. And this miR159-MYB81 module shows opposing expression patterns (Table S8). These results indicate that the downregulation of miR159 in maize might participate in an HT stress-related signaling pathway and thereby contribute to defensive response to HT stress.

In *Arabidopsis*, miR166a is known to target mRNAs encoding HD-Zip TFs [58], which have been shown to be required for the establishment of the apical meristem, proper formation pattern in lateral organs, and leaf development [59]. In this study, two upregulated targets (Zm00001d013699_T001 and Zm00001d048527_T027), encoding the HD-Zip TFs ATHB-14 and REVOLUTA, respectively, showed the opposite regulation with respect to their cognate miR166 (Table S8). The REVOLUTA gene is necessary for apical meristem development and for limiting cell divisions in the leaves and stems of *Arabidopsis* [60]. Thus, the downregulation of miR166 might influence the leaf morphology through the modulation of HD-Zip TFs under HT stress.

Plant growth regulating factor (GRF) genes encode a family of putative transcription factors that are generally recognized as target genes of miR396 and have been reported to play roles in plant leaf growth and the responses to various environmental stresses [61,62], not including HT stress. In present study, the miR396–GRF module was also identified and the expression of genes encoding GRF TFs and miR396 was upregulated and downregulated, respectively, which suggested that the miR396–GRF module might also be involved in maize leaf growth under HT stress.

SAC2 is one of the phosphoinositide phosphatases participating in inositol phosphate metabolism, the phosphatidylinositol signaling system and the endocytic pathway [63] and is related to the stress response [64]. In our study, the upregulated gene (Zm00001d048258) encoding phosphoinositide phosphatase SAC2 was targeted by the downregulated sbi-miR5381_L-1R+3, which indicated that miR5381 is involved in signaling transduction under HT stress. Moreover, another upregulated gene encoding vacuolar protein sorting-associated protein 24 (VPS24) homolog 1 was targeted by the downregulated miRn202 (Table S8) and VPS24 homolog 1 was enriched in vacuolar transport and the endocytosis pathway, which is consistent with a recent report that identified it as one subunit of the *Arabidopsis* phosphatidylinositol-3-kinase complex, in which the VPS38 plays critical roles in autophagy and endosome sorting [65].

## 4. Materials and Methods

### 4.1. Plant Material, Heat Treatment, and Sample Collection

*Zea mays* (maize) inbred line 78599-3, a male parent of the heat-sensitive maize hybrid Zhuyu 309, was pot-planted in the farm of Henan Agricultural University, Zhengzhou (Henan Province, China). When they reached the V_8_ stage, healthy and uniform plants were selected and randomly divided into two groups. One group was moved into a growth chamber with a 30/25 °C (day/night) temperature as the control (CK). The other group was moved into a growth chamber with a 40/25 °C (day/night) as HT stress. Both groups were grown under a 60% humidity, a 12/12 h (day/night) photoperiod, and a photosynthetic photon flux density of 1000 μmol m^−2^ s^−1^. Except for the main vein, the middle part of the 10th leaf from individual plants under CK and HT conditions was collected five days after treatment, frozen immediately in liquid nitrogen, and stored at −80 °C. Leaves from three individual plants grown under CK or HT conditions were pooled as one replicate, with three replicates for each group.

### 4.2. Determination of Physiological Parameters of Maize Leaves

Chlorophyll and carotenoid content were measured in 80% acetone extract using ultraviolet (UV) spectrophotometry as described previously [66], with minor modification. Briefly, 0.2 g leaves were collected and cut into pieces before soaking with 20 mL 80% acetone extract. The extract with leaves was stored in the dark at room temperature for 24 h. Chlorophyll and carotenoid concentrations were measured spectrophotometrically at 663, 645, and 470 nm, respectively.

The rate of O_2_^−^ production was measured by monitoring the nitrite formation from hydroxylamine in the presence of O_2_^−^ following the method described by [67]. In brief, 1 g leaf segments were homogenized with 3 mL of 65 mM potassium phosphate buffer (pH 7.8) and centrifuged at 5000× *g* for 10 min. The incubation mixture contained 0.9 mL of 65 mM phosphate buffer (pH 7.8), 0.1 mL of 10 mM hydroxylamine hydrochloride, and 1 mL of the supernatant. After incubation at 25 °C for 20 min, 58 mM sulfanilamide and 7 mM α-naphthylamine were added to the incubation mixture. After reaction at 25 °C for 20 min, ethyl ether in the same volume was added and centrifuged at 1500× *g* for 5 min. The absorbance in the aqueous solution was read at 530 nm. A standard curve with NO_2_^−^ was used to calculate the production rate of O_2_^−^ from the chemical reaction of O_2_^−^ and hydroxylamine. The content of H_2_O_2_ was measured by monitoring the A_415_ of the titanium-peroxide complex according to previous description [68]. Absorbance values were calibrated to a standard curve generated with known concentrations of H_2_O_2_.

Lipid peroxidation was determined by measuring the amount of malondialdehyde (MDA) produced by the thiobarbituric acid reaction according to previous description [69]. In brief, leaves were homogenized in 20% (*w*/*v*) trichloroacetic acid (TCA). The supernatant was then mixed with equal volume 0.5% (*w*/*v*) thiobarbituric acid and heated for 15 min in a 95 °C water bath. The mixture was centrifuged at 3000× *g* for 10 min and the absorbance of the supernatant was monitored at 532 and 600 nm. After subtracting the non-specific absorbance (600 nm), the MDA concentration was determined by its molar extinction coefficient 155 mM^−1^ cm^−1^) and the results expressed as mmol MDA g^−1^ FW.

For assay of antioxidant enzyme activities, total superoxide dismutase (SOD), catalase (CAT) and peroxidase (POD) activity of maize leaves was determined as described previously [70]. Briefly, 0.2 g leaves were homogenized in a mortar and pestle with 4 mL of ice-cold extraction buffer (100 mM potassium phosphate buffer, pH 7.0, 100 μM EDTA). The homogenate was centrifuged at 16,000× *g* for 20 min. The supernatant fraction was used as crude extract for enzyme activity. Total SOD activity was determined by measuring its ability to inhibit the photochemical reduction of nitro blue tetrazolium chloride (NBT). Total CAT activity was measured according to the decrease of H_2_O_2_ monitored at 240 nm and quantified by its molar extinction coefficient (36 mM^−1^ cm^−1^). The result was expressed as mmol H_2_O_2_ min^−1^ g^−1^ FW. Total POD activity was determined by following the decrease in ascorbate at 290 nm (extinction coefficient 2.8 mM^−1^ cm^−1^) for 1 min and the results expressed in mmol H_2_O_2_ min^−1^ g^−1^ FW, taking into consideration that 2 mol ascorbate are required for reduction of 1 mol H_2_O_2_.

### 4.3. RNA Isolation, sRNA Library Preparation and Sequencing

Total RNA was extracted from the CK and HT leaves with three biological replicates using TRIzol reagent (Invitrogen, Carlsbad, CA, USA) according to the manufacturer’s instructions. The quality and purity of the total RNA was analyzed using Bioanalyzer 2100 and an RNA 6000 Nano LabChip Kit (Agilent, Santa Clara, CA, USA). Approximately 1 μg of total RNA was used for each sRNA library preparation according to the TruSeq Small RNA Sample Prep Kit protocol (Illumina, San Diego, CA, USA). In brief, 1 μg of total RNA was ligated to RNA-DNA chimeric oligonucleotide adaptors and converted to cDNA by RT-PCR. The resulting cDNA was amplified by PCR and gel-purified for the construction of sequencing libraries. Finally, sRNA sequencing (single-end, 50 bp) was performed on an Illumina Hiseq2500 platform (LC-BIO, Hangzhou, China) according to the manufacturer’s recommended protocol.

### 4.4. Identification of Known and Novel miRNAs

The raw reads were processed to remove adapters and junk sequences using Illumina’s Genome Analyzer Pipeline. Subsequently, the sequences matching mRNA, rRNA, tRNA, snRNA, snoRNA and repeats were classified and filtered by alignment to NCBI databases, Rfam (http://rfam.janelia.org) and Repbase (http://www.girinst.org/repbase) using Bowtie, an alignment software in the proprietary pipeline script ACGT101-miR (LC Sciences, Houston, TX, USA). The remaining unique reads with length in 18~25 nt were aligned against miRBase (http://www.mirbase.org) and maize genome (ftp://ftp.ensemblgenomes.org/pub/plants/release-37/fasta/zea_mays/dna/Zea_mays.AGPv4.dna) using bowtie to identify known and potential novel miRNAs. Length variation at both 3’ and 5’ ends and one mismatch inside of the sequence were allowed in the alignment. Based of sequence similarity to maize genome, these miRNA sequences were classified into subgroups gp1a, gp1b, gp2 and gp4 according to the following criteria: reads that were mapped to maize pre-miRNAs and whose pre-miRNAs were further mapped to maize genome were classified as gp1a; reads that were mapped to pre-miRNAs of selected species (with the exception of maize) in miRbase and whose pre-miRNAs were further mapped to maize genome were classified as gp1b; reads that were mapped to pre-miRNAs of selected species (with the exception of maize) and maize genome but whose pre-miRNAs were not further mapped to maize genome, were classified as gp2; reads that were not mapped to any pre-miRNAs of selected species but mapped to maize genome, and whose predicted hairpins formed by their extended genome sequences were further aligned to maize genome were classified as gp4. Among those subgroups, gp1a, gp1b and gp2, and gp4 were identified as known and novel miRNAs, respectively. The secondary structures of pre-miRNAs were predicted by Mfold software (http://unafold.rna.albany.edu/?q=mfold/download-mfold) based on the key criteria for miRNA prediction [20]. Based on normalized deep-sequencing counts, miRNA differential expression was analyzed using Student’s *t*-test. The significance threshold was set to 0.05 in this test.

### 4.5. Degradome Sequencing and Target Identification

Equal amounts of six total RNA samples were mixed together. Approximately 20 μg of the pooled total RNA sample was used to generate one degradome library. The mRNA was mixed with biotinylated random primers, and then the RNA containing the biotinylated random primers was captured by beads and ligated to 5′ adaptors. Purified ligated products were reverse-transcribed to first-strand cDNA and then amplified with PCR. Following purification, digestion, ligation and repurification, the cDNA library was sequenced (single-end, 36 bp) with an Illumina Hiseq2500 (LC-BIO, Hangzhou, China). The extracted sequencing reads were then used to identify potentially cleaved targets by the CleaveLand3.0 pipeline [71]. The degradome reads were mapped to the mRNAs of maize (ftp://ftp.ensemblgenomes.org/pub/plants/release-37/fasta/zea_mays/). Only perfectly matching alignments for a given read were kept for degradation analysis. Targets of the miRNAs were identified by TargetFinder.

### 4.6. mRNA Library Construction and Sequencing

The total RNA that had been used for sRNA library construction was also used for mRNA library preparation. Briefly, approximately 15 μg of total RNA was used to generate each mRNA library using an Illumina TruSeq RNA Sample Preparation Kit (Illumina, San Diego, CA, USA) and mRNA sequencing (paired-end, 150 bp) was carried out on an Illumina Hiseq4000 at LC-BIO (Hangzhou, China) following the vendor’s recommended protocol. Clean reads were obtained by removing the adapters and low-quality reads from the raw data. HISAT-2.0 was used for mapping the reads to the maize reference genome and StringTie-1.3.0 tool was used for assembling transcripts and estimating their abundances by calculating FPKM values [72]. The mRNAs and genes with an absolute value of log_2_ (FC) >1 and *p* value < 0.05 identified by the Ballgown package in R software were deemed to be differentially expressed. GO and KEGG enrichment analysis of the DEGs was then performed using R package-clusterProfiler [73].

### 4.7. Verification of RNA-seq Data by qRT-PCR

To test the reliability of RNA-seq data, six miRNAs and their target genes were selected for qRT-PCR. Specific primers were designed with the Primer Express software (Applied Biosystems, Waltham, MA, USA). cDNA was synthesized from 1 μg of total RNA using the PrimeScript RT reagent Kit (Takara, Dalian, China). The cDNA of miRNA was generated by stem-loop RT-PCR using miRNA-specific stem-loop primers, while cDNA of mRNA was generated based on Oligo dT primer. Real-time RT-PCR was performed on the ABI 7500 Real-Time PCR System using the 2× SYBR green PCR master mix (Applied Biosystems, Waltham, MA, USA). A maize housekeeping gene, 18S rRNA, served as the internal control for real-time RT-PCR reactions. The relative expression of all mRNAs and miRNAs were calculated using 2^−ΔΔ*C*t^ method. Three biological replicates were performed for each group, and data were indicated as mean ± SE (*n* = 3). All primers used for qRT-PCR are shown in Table S10.

## 5. Conclusions

In summary, by far we firstly performed an integrated analysis of small RNA, RNA and degradome sequencing of maize during exposure to HT stress to generate a comprehensive resource focused on the identification of key regulatory miRNA-target modules in maize under HT stress, and identified 102 miRNAs of maize in response to HT stress, including 61 known and 41 novel miRNAs. In this study, it was revealed that a total of 5982 genes were found to be significantly differentially expressed and that the target genes for HT-responsive miRNAs mainly functioned in development process, plasma membrane system, signal transduction and modulation of ROS and leaf senescence. Furthermore, 30 miRNA–mRNA pairs showing significant inverse expression patterns were screened through the integrated analysis and these interaction pairs might be the key miRNA-mRNA regulatory modules in maize under HT stress. Our findings provide an experimental foundation for elucidating the miRNA-mediated post-transcriptional regulation of maize in response to HT stress. Further transgenic confirmation of these identified interaction pairs will provide more solid information.

## Figures and Tables

**Table 1:** Identified target transcription factors of known DEMs by degradome analysis.

miR_family	Targets Annotation	TF Family
miR1430	Nuclear transcription factor Y subunit A-10	NF-YA
miR156	neighbor of tga1	SBP/SPL
	SBP-domain containing protein	
	SBP-domain protein 5	
	Squamosa promoter-binding-like protein 11	
	Squamosa promoter-binding-like protein 15	
	Squamosa promoter-binding-like protein 18	
	Squamosa promoter-binding-like protein 2	
	Squamosa promoter-binding-like protein 5	
	Squamosa promoter-binding-like protein 6	
	tassel sheath4	
	teosinte glume architecture1	
miR160	Auxin response factor 16	ARF
miR164	NAC domain-containing protein 21/22	NAC
	NAC domain-containing protein 79	
miR166	Homeobox-leucine zipper protein ATHB-14	HD-ZIP
	Homeobox-leucine zipper protein ATHB-14	
	Homeobox-leucine zipper protein REVOLUTA	
	rolled leaf2	
miR169	CCAAT-HAP2-transcription factor 26	NF-YA
	nuclear transcription factor y subunit a1	
	Nuclear transcription factor Y subunit A-10	
	Nuclear transcription factor Y subunit A-3	
	Nuclear transcription factor Y subunit A-8	
	Nuclear transcription factor Y subunit A-9	
	SBP-transcription factor16	SBP
miR172	Floral homeotic protein APETALA 2	AP2
	Putative AP2/EREBP transcription factor superfamily protein	
	sister of indeterminate spikelet1	
	tasselseed6	
miR396	Growth-regulating factor	GRF
	Growth-regulating factor 2	
	Growth-regulating factor 6	
	Putative growth-regulating factor 6	

**Table 2:** GO category of targets of known DEMs identified by degradome.

GO ID	Ontology	Description	Gene Ratio
GO:0006351	BP	transcription, DNA-templated	54/323
GO:0032502	BP	developmental process	30/323
GO:0006816	BP	calcium ion transport	18/323
GO:0070588	BP	calcium ion transmembrane transport	18/323
GO:0007275	BP	multicellular organism development	14/323
GO:0009725	BP	response to hormone	12/323
GO:0009734	BP	auxin-activated signaling pathway	10/323
GO:0055114	BP	oxidation-reduction process	5/323
GO:0006508	BP	proteolysis	4/323
GO:0005634	CC	nucleus	141/323
GO:0016020	CC	membrane	40/323
GO:0005737	CC	cytoplasm	31/323
GO:0005783	CC	endoplasmic reticulum (ER)	1/323
GO:0003677	MF	DNA binding	142/323
GO:0008289	MF	lipid binding	51/323
GO:0003700	MF	transcription factor activity	47/323
GO:0042578	MF	phosphoric ester hydrolase activity	21/323
GO:0005388	MF	calcium-transporting ATPase activity	18/323
GO:0003824	MF	catalytic activity	9/323
GO:0016491	MF	oxidoreductase activity	5/323

**Table 3:** GO category of targets of novel DEMs identified by degradome.

GO ID	GO Ontology	Description	Gene Ratio
GO:0006629	BP	lipid metabolic process	5/19
GO:0006468	BP	protein phosphorylation	5/19
GO:0016310	BP	phosphorylation	5/19
GO:0006139	BP	nucleobase-containing compound metabolic process	1/19
GO:0090305	BP	nucleic acid phosphodiester bond hydrolysis	1/19
GO:0006412	BP	translation	1/19
GO:0055085	BP	transmembrane transport	1/19
GO:0016021	CC	integral component of membrane	9/19
GO:0016020	CC	membrane	9/19
GO:0005840	CC	ribosome	1/19
GO:0005622	CC	intracellular	1/19
GO:0008889	MF	glycerophosphodiester phosphodiesterase activity	5/19
GO:0008081	MF	phosphoric diester hydrolase activity	5/19
GO:0004672	MF	protein kinase activity	5/19
GO:0016301	MF	kinase activity	5/19
GO:0005524	MF	ATP binding	5/19
GO:0016740	MF	transferase activity	2/19
GO:0008408	MF	3′-5′ exonuclease activity	1/19
GO:0019706	MF	protein-cysteine S-palmitoyltransferase activity	1/19
GO:0022857	MF	transmembrane transporter activity	1/19

**Table 4:** List of the miRNA-mRNA pairs related to vital pathway.

miRNA ID	log2(FC)	pval	Transcript ID	log2(FC)	pval	Pathway
osa-MIR5079a-p3_2ss2CT21TA	6.3	0.01	Zm00001d027612_T003	−1.9	0.03	Porphyrin and chlorophyll metabolism
						Aminoacyl-tRNA biosynthesis
miRn218	3.4	0.01	Zm00001d016463_T005	−3.7	0.02	Steroid biosynthesis
			Zm00001d022429_T030	−12.6	0.00	RNA degradation
			Zm00001d022429_T036	−12.6	0.00	
miRn242	−0.9	0.00	Zm00001d051080_T001	4.6	0.00	Purine metabolism
			Zm00001d051080_T006	4.3	0.00	
			Zm00001d051080_T009	4.3	0.00	
			Zm00001d002141_T002	2.2	0.03	Spliceosome
zma-miR164f-5p	−1.0	0.04	Zm00001d043152_T001	4.7	0.02	Pyrimidine metabolism
						beta-Alanine metabolism
						Pantothenate and CoA biosynthesis
zma-miR159a-5p	−1.1	0.01	Zm00001d029550_T013	9.3	0.00	Glycerolipid metabolism
						Glycerophospholipid metabolism
						Phosphatidylinositol signaling system
miRn248	−1.4	0.01	Zm00001d043998_T004	3.1	0.00	Protein processing in ER
						Ubiquitin mediated proteolysis
miRn194	−1.6	0.04	Zm00001d002141_T002	2.2	0.03	Spliceosome
miRn202	−2.3	0.01	Zm00001d027314_T002	1.6	0.02	Endocytosis

## Supplementary Materials

Supplementary materials can be found at https://www.mdpi.com/1422-0067/20/7/1754/s1.

## Abbreviations

HT	High Temperature
ROS	Reactive Oxidative Species
SOD	Superoxide Dismutase
CAT	Catalase
POD	Peroxidase
MDA	Malonaldehyde
TF	Transcription Factor
GRF	Growth Regulation Factor
qRT-PCR	Quantitative Real-Time PCR
AP2/EREBP	APETALA2/Ethylene-Responsive Element Binding Protein
